# Supplementary material for: Proton-assisted creation of controllable volumetric oxygen vacancies in ultrathin CeO2−x for pseudocapacitive energy storage applications
Source: Nat Commun. 2019 Jun 13;10:2594. doi: 10.1038/s41467-019-10621-2 (PMC6565713; doi:10.1038/s41467-019-10621-2)
Supplement: Supplementary file 1 — Supplementary Information [file 41467_2019_10621_MOESM1_ESM.pdf]

Supplementary Information for

Proton-Assisted Creation of Controllable Volumetric Oxygen Vacancies in Ultrathin CeO_{2-x} for Pseudocapacitive Energy Storage Application

Mofarah *et al*

Table of Contents

Supplementary Note	Titles	Page
1	Thermodynamic analyses of Ce(III) in Ce^{3+}-Ce^{4+}-CH_3COOH-H_2O system	3
2	Cyclic voltammetry analyses of Ce(III)/Ce(IV) species in Ce^{3+}-Ce^{4+}-CH_3COOH-H_2O system	5
3	Characterisation of ultrathin CeO_{2-x} films	9
4	Quantitative XPS and EELS analyses	10
5	Effect of pH on electrodeposition of CeO_{2-x}	11
6	Chemical identification and calculation of peak areas observed in cyclic voltammetry deposition of CeO_{2-x} thin films	13
7	Mechanism of electrodeposition of CeO_{2-x} (calculation of Gibbs free energy required for $V_O^{\bullet\bullet}$ formation (ΔG_{Vo}))	15
8	Energy storage applications	17
9	Effect of crystallite size on calculated band gap energies	18
10	Effect of scan rate on pseudocapacitive behaviour	19
11	Effect of cycle number on redox pseudocapacitive behaviour	23
12	Comparison with other work	26
13	Computational methodology for calculation of $Ce(OH)_4$ Gibbs free energy	26

Supplementary Note 1. Thermodynamic analyses of Ce(III) in Ce^{3+}-Ce^{4+}-CH_3COOH-H_2O system

The thermodynamic behaviour of aqueous Ce(III) and Ce(IV) species was studied in Ce^{3+}-CH_3COOH-H_2O, Ce^{4+}-CH_3COOH-H_2O, and Ce^{3+}-Ce^{4+}-CH_3COOH-H_2O systems; the data are given in Figure 1. Thermodynamic analyses were done using MEDUSA, the associated database HYDRA (Hydrochemical Equilibrium Constant Database), and HSC software. For most species, the Gibbs free energies in the HYDRA database were substituted by more recent values from Hayes *et al.*[1], Chirkst *et al.*[1,2], Channei *et al.*[3], and Lange's Chemistry Handbook[4]. In the case of absent values, these were calculated by density functional theory (DFT) (simulation methodology in Supplementary Note 13). The calculations were done according to standard-state conditions of T (temperature) = 298 K and P (pressure) = 1 atm. The initial concentration of both $Ce(NO_3)_3{\cdot}6H_2O$ and $Ca(CH_3COOH)_2$ was selected to be 0.05 M according to the experimental conditions. The associated speciation diagrams, which plot the concentrations of soluble species as a function of pH are shown in Figure 1(a-c) and the Pourbaix diagram mapping the predominant species, is shown in Figure 1(d). Precipitated $Ce(OH)_{3(pt)}$, which forms at pH 7.8, has been omitted for simplification of the analysis. Speciation and Pourbaix diagrams that include $Ce(OH)_{3(pt)}$ have been published elsewhere[3]. In Figure 1(a), Ce^{3+} is the predominant Ce species at pH <4.6 owing to the intrinsic high dissociation constant of CH_3COOH, which causes its concentration to decrease slowly and so release gradually the conjugate base of CH_3COO^- below the given pH. This predominance competes with the rapid deprotonation of CH_3COOH at higher pH values, which establishes $Ce(CH_3COO)^{2+}$ as the predominant Ce species up to pH ~9.1. At greater pH values, the increasing influence of the field strength of Ce^{3+} results in the sequential predominance of $\mathrm{Ce(OH)_2^+}$ and then aqueous $Ce(OH)_{3(aq)}$. Figure 1(b) shows the predominant species of aqueous Ce(IV), the high field strength of which favours hydroxylation rather than forming a conjugate pair with CH_3COO^-. Figure 1(c) shows that the hydroxylation is limited to pH < ~1.5, above which the precipitation of $Ce(OH)_{4(pt)}$ suppresses the presence of aqueous $Ce(OH)_{4(aq)}$. In the Pourbaix diagram of Figure 1(d), the solid black lines delineate the predominance regions for the Ce species. The central light region enclosed by two dashed lines defines the conditions of reduction potential (E^0 *vs.* HSE electrode) and pH, where water is stable and no oxygen or hydrogen evolution occurs. At theoretical pH < 3.7, the formation of Ce(IV) species is suppressed by water oxidation, where oxygen bubbling on the substrate impedes the direct oxidation of Ce^{3+} cations. At higher pH, the potential required for Ce(III) oxidation is within the water stability region.

Thermodynamic studies of cerium in aqueous solution have been reported previously[1,5,6]. In these, the Gibbs free energy of formation (ΔG_f^0) for $Ce(OH)_{4(aq)}$ was considered to be the product of the oxidation of Ce(III) species. Further, the effect of ionic concentration and strength in the calculations

of the theoretical redox potentials were not considered, which have led to misconceptions concerning the electrochemical characteristics of cerium in aqueous solutions. Factoring these considerations into the present experimental work demonstrates that the oxidation of Ce(III) species causes the precipitation of $Ce(OH)_{4(pt)}$. Since these species have received little attention, the only calculated data reported[7] have been confirmed by DFT simulation of the ΔG_f^0 (methodology in Supplementary Note 13). The Pourbaix diagram also differentiates between what are effectively the stability regions for $Ce(OH)_{4(aq)}$ (area for pH>2.8, which is enclosed with solid (predominance) and dotted (water stability) black lines) and $Ce(OH)_{4(pt)}$ (red); the relationship between these and $CeO_2{\cdot}2H_2O$ will be discussed subsequently.

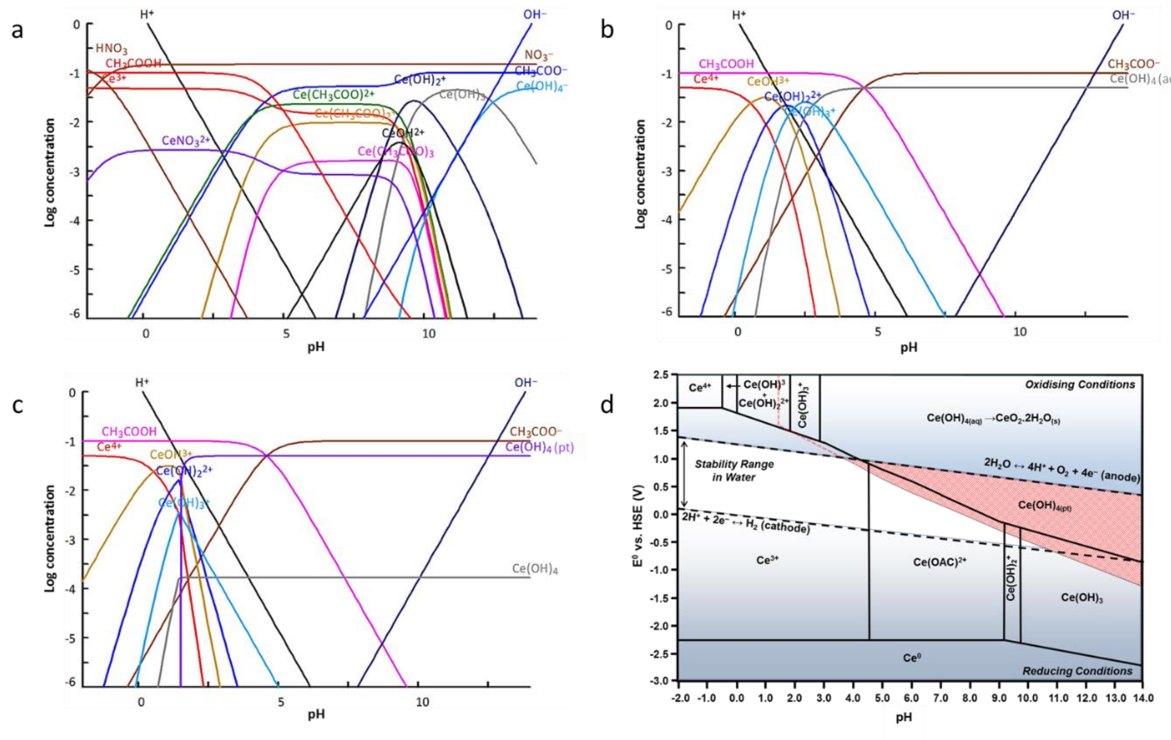

Supplementary Figure 1. Speciation diagrams for relevant systems. (a) Ce^{3+}-CH_3COOH-H_2O, (b) Ce^{4+}-CH_3COOH-H_2O without $Ce(OH)_4$ precipitate, (c) Ce^{4+}-CH_3COOH-H_2O with $Ce(OH)_4$ precipitate systems, and (d) Pourbaix diagram of Ce^{3+}-Ce^{4+}-CH_3COOH-H_2O (for simplification, $Ce(OH)_{3(pt)}$ is omitted).

Since the Pourbaix diagram presents only the stability regions of predominant species, then extensive thermodynamic calculations were conducted in order to obtain theoretical redox potentials for all of the possible Ce(III) species. The corresponding data are plotted against pH values, as shown in Figure 2.

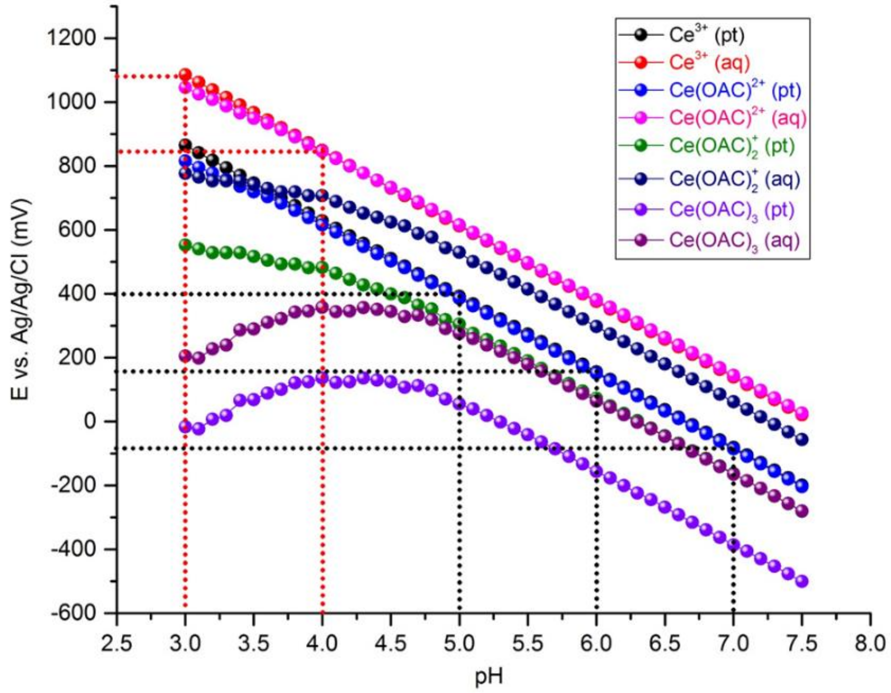

Supplementary Figure 2. Potential variations for redox reactions of Ce-CH_3COO-H_2O system corresponding to aqueous (aq) and precipitate (pt) Ce(III) species.

Supplementary Note 2. Cyclic voltammetry analyses of Ce(III)/Ce(IV) species in Ce^{3+}-Ce^{4+}-CH_3COOH-H_2O system

The electrochemical behaviour of the Ce(III) ↔ Ce(IV) redox reactions was studied using cyclic voltammetry for the Ce^{3+}-Ce^{4+}-CH_3COOH-H_2O system at various scan rates (υ) and pH values, as shown in Figure 3. Figure 3(a) shows the voltammograms obtained at pH = 3 for υ = 20-1000 mVs^{-1}. At slow υ = 20 mVs^{-1}, where the electrolyte resistance is minimal, the anodic peak for oxidation of Ce(III) species occurs at E_{pa} = 1175 mV, while the reverse cathodic peak for reduction occurs at E_{pc} = 1003 mV. The redox potential ($E_{1/2}$) was integrated to be +1089 mV, being close to the theoretical potential for the $Ce^{3+} \leftrightarrow Ce(OH)_{4(aq)}$ reaction ($E_{1/2}$ = +1085 mV given in Table 1). Comparison of the experimental and calculated $E_{1/2}$ for $3 \leq pH \leq 7$ are given in Table 1-4.

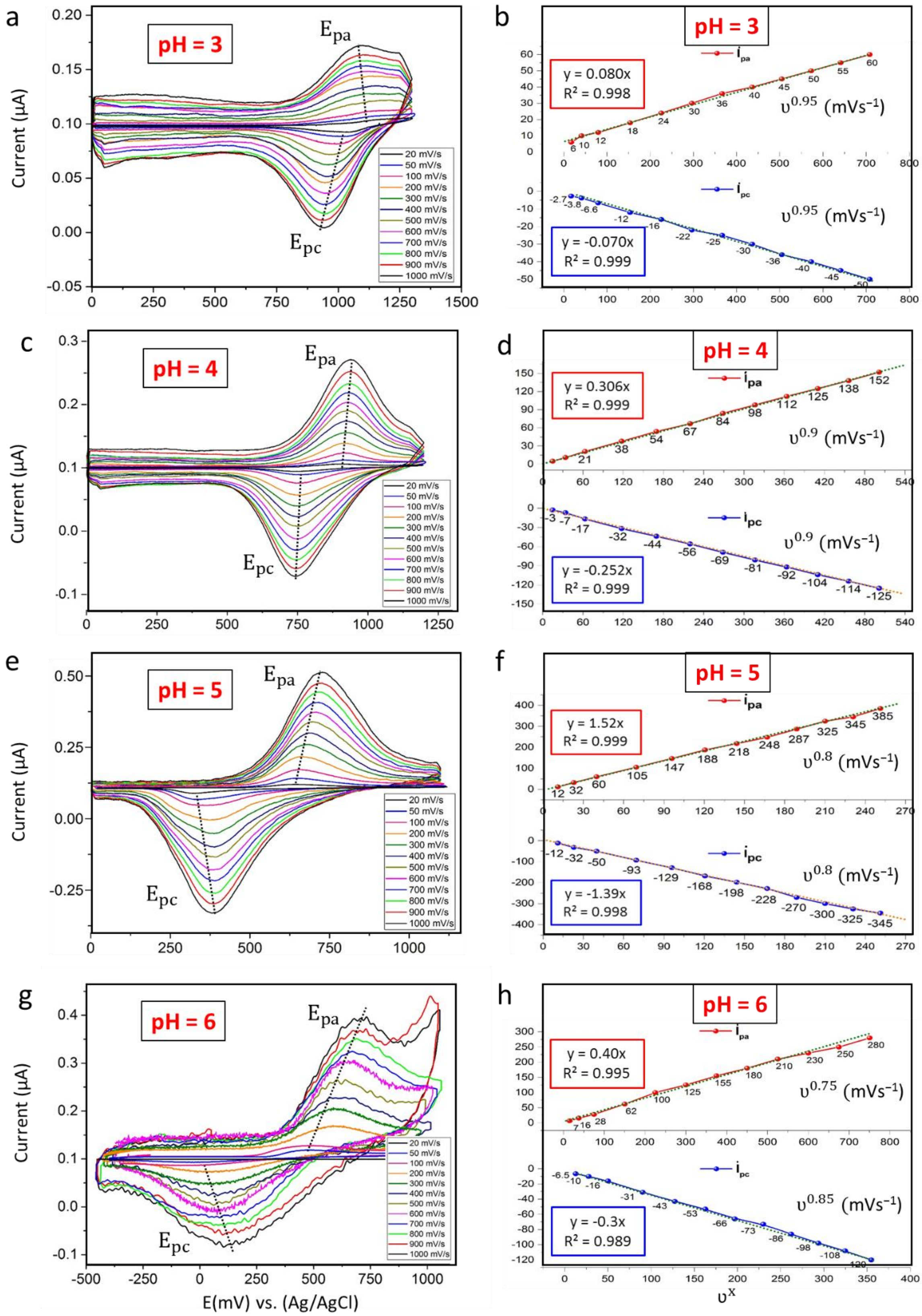

Supplementary Figure 3. Cyclic voltammetry analyses of Ce-CH_3COO-H_2 system at υ = 20-1000 mVs^{-1} and different pH: (a) 3 (b) 4, (c) 5, (d) 6. Plot of peak current (I_p) as a function of scan rate (υ) obtained from cyclic voltammetry analysis at pH: (e) 3 (g) 4, (g) 5, (h) 6.

Considering the $E_{1/2}$ at pH = 3, although the Pourbaix diagram of Figure 1(d) shows that $Ce(OH)_{4(pt)}$ is thermodynamically more stable (as reflected by the E) than $Ce(OH)_{4(aq)}$, the experimental data are contradictory in that $Ce(OH)_{4(aq)}$ forms rather than the precipitate. This is due to that oxidation of the Ce^{3+} to Ce^{4+} involves generation of protons, which consequently decreases the local pH and make a significant shift of the composition towards the stability of $Ce(OH)_{4(aq)}$.

The kinetics analysis of the redox reaction at pH = 3 was carried out by measuring the cathodic (i_{pc}) and anodic peak currents (i_{pa}) of the cyclic voltammograms (Figure 3a). In order to determine the controlling mechanisms of redox processes, the relation of i_{pc} and i_{pa} with υ^x ($0.5 \leq x \leq 1$) is plotted in Figure 3(b). The best linear fit with zero intercept was obtained at x = 0.95 for both oxidation and reduction reactions, confirming that the redox mechanism is controlled almost by surface confinement and negligible role of ionic species diffusion[8]. Further on kinetics of the redox reaction, the peak potentials (ΔE_p) were assessed in order to determine the degree of reversibility of the $Ce^{3+} \leftrightarrow Ce(OH)_4$ reaction. As shown in Figure 3(a), raising the υ results in successive negative shifts in peak potentials, while the ΔE_p values remain constant at 130 mV. This indicates an independence nature of ΔE_p from υ.

Although the constant ΔE_p value, as a function of υ, illustrates high reversibility degree of $Ce^{3+} \leftrightarrow Ce(OH)_4$ reaction at acidic pH = 3, according to Bockris[9] theory, where ΔE_p should be ≤26 mV for a fully reversible reaction, such redox reaction is considered as thermodynamically irreversible.

Figure 3(c) shows the voltammograms obtained at pH = 4 for υ = 20-1000 mVs^{-1}. The $E_{1/2}$ = +825 mV is consistent with the theoretical $E_{1/2}$ corresponding to $Ce^{3+} \leftrightarrow Ce(OH)_{4(aq)}$ and/or $Ce(CH_3COO)^{2+} \leftrightarrow Ce(OH)_{4(aq)}$ (see Tables 1 and 2). Similar to situation at pH = 3, $Ce(OH)_{4(aq)}$ formation occurs. Increasing the pH from 3 to 4 further decreases reversibility degree of the redox reactions, as indicated by the increased ΔE_p of 152 mV at υ = 20 mVs^{-1}. This effect is amplified since ΔE_p increased to 196 mV upon increasing the υ to 1000 mVs^{-1}. The controlling mechanisms of redox processes was also measured by plotting the i_{pc} and i_{pa} against υ^x ($0.5 \leq x \leq 1$). The best linear fit with zero intercept for the redox reactions at pH = 4 was obtained at x = 0.9 (Figure 3(d)), illustrating that the predominant mechanism is surface confinement, while its contribution is reduced by 5%.

At pH = 5, the voltammograms shown in Figure 3(e) reveal a $E_{1/2}$ of +455 mV at υ = 20 mVs^{-1}, which is close to those calculated for $Ce^{3+} \leftrightarrow Ce(OH)_{4(pt)}$ and $Ce(CH_3COO)^{2+} \leftrightarrow Ce(OH)_{4(pt)}$. The increasing ΔE_p values reach 340 mV, being greater than that obtained at pH = 4 (152 mV). As shown in Figure 3(f),

linear fitting of the peak currents against log υ^x at zero intercept yields an exponent of x = 0.8, confirming that increasing pH is associated with increasing contribution from diffusion, although surface confinement still remains dominant. Consequently, increasing the pH gradually reduces the degree of reversibility, thereby slowing the kinetics of the redox reaction.

According to the Nernst equation[10], $E_{1/2}$ values for Ce(III)-involved reactions were calculated, the plot of which against pH is shown in Figure 2. As can be seen, increasing the pH by 1 unit is expected to decrease the $E_{1/2}$ by 236 mV. This originates to the nature of cerium redox reaction that involves an electron and four protons (ignoring the minor effect of the species concentration). The experimentally determined $E_{1/2}$ matches those calculated except at 4 ≤ pH ≤ 5, over which range the decrease was significantly larger. Such contradiction can be ascribed to two phenomenon: 1) variations in the types of Ce(III) species involved in the redox reactions, which is determined by the speciation diagrams, and 2) shifting the redox reaction toward the precipitation or re-dissolution of $Ce(OH)_{4(pt)}$. The nature of the redox reaction as a function of pH is determined by matching the experimental $E_{1/2}$ with the calculated values, the plots of which are shown in Figure 2.

At pH = 6, the voltammograms shown in Figure 3(g) indicates a $E_{1/2}$ of +210 mV at υ = 20 mVs^{-1}, which correspond to either $Ce(CH_3COO)^{2+} \leftrightarrow Ce(OH)_{4(pt)}$ and $Ce^{3+} \leftrightarrow Ce(OH)_{4(pt)}$. The continuing retardation of the kinetics is confirmed by increased ΔE_p of 615 mV and decreased exponent of x = 0.75 for oxidation reaction, as shown in Figure 3(h). However, the cathodic reaction shows superior kinetics of reactions, compared to the oxidation, with the exponent of x = 0.85.

Supplementary Table 1. Redox potentials ($E_{1/2}$) for reaction of Ce^{3+} to form $Ce(OH)_4$ or $CeO_2{\cdot}2H_2O$ (green font for agreement between experimental (red font) and calculated (black font) values).

pH	Experimental redox potential (V)	Calculated $E_{1/2}$ for reaction between Ce^{3+} and $Ce(OH)_{4(pt)}$	Calculated $E_{1/2}$ for reaction between Ce^{3+} and $Ce(OH)_{4(aq)}$	Calculated $E_{1/2}$ for reaction between Ce^{3+} and $CeO_2{\cdot}2H_2O$
3	+1.089	+0.865	+1.085	+0.355
4	+0.825	+0.629	+0.849	+0.119
5	+0.455	+0.392	+0.612	–0.117
6	+0.210	+0.157	+0.376	–0.354
7	–0.019	–0.080	+0.139	–0.590

Supplementary Table 2. Redox potentials ($E_{1/2}$) for reaction of $Ce(CH_3COO)^{2+}$ to form $Ce(OH)_4$ or $CeO_2{\cdot}2H_2O$ (green font) for agreement between experimental (red font) and calculated (black font) values).

pH	Experimental redox potential (V)	Calculated $E_{1/2}$ for reaction between $Ce(CH_3COO)^{2+}$	Calculated $E_{1/2}$ for reaction between	Calculated $E_{1/2}$ for reaction between

		and $Ce(OH)_{4(pt)}$	$Ce(CH_3COO)^{2+}$ and $Ce(OH)_{4(aq)}$	$Ce(CH_3COO)^{2+}$ and $CeO_2{\cdot}2H_2O$
**3**	+1.089	+0.815	+1.046	–0.314
**4**	+0.825	+0.615	+0.845	+0.113
**5**	+0.455	+0.385	+0.615	–0.116
**6**	+0.210	+0.152	+0.381	–0.350
**7**	–0.019	–0.085	+0.144	–0.587

Supplementary Table 3. Redox potentials ($E_{1/2}$) for reaction of $Ce(CH_3COO)_2^+$ to form $Ce(OH)_4$ or $CeO_2{\cdot}2H_2O$.

pH	Experimental redox potential (V)	Calculated $E_{1/2}$ for reaction between $Ce(CH_3COO)_2^+$ and $Ce(OH)_{4(pt)}$	Calculated $E_{1/2}$ for reaction between $Ce(CH_3COO)_2^+$ and $Ce(OH)_{4(aq)}$	Calculated $E_{1/2}$ for reaction between $Ce(CH_3COO)_2^+$ and $CeO_2{\cdot}2H_2O$
**3**	+1.089	+0.552	+0.777	+0.052
**4**	+0.825	+0.482	+0.707	–0.018
**5**	+0.455	+0.304	+0.530	–0.195
**6**	+0.210	+0.072	+0.297	–0.427
**7**	–0.019	–0.162	+0.062	–0.662

Supplementary Table 4. Redox potentials ($E_{1/2}$) for reaction of $Ce(CH_3COO)_3$ to form $Ce(OH)_4$ or $CeO_2{\cdot}2H_2O$.

pH	Experimental redox potential (V)	Calculated $E_{1/2}$ for reaction between $Ce(CH_3COO)_3$ and $Ce(OH)_{4(pt)}$	Calculated $E_{1/2}$ for reaction between $Ce(CH_3COO)_3$ and $Ce(OH)_{4(aq)}$	Calculated $E_{1/2}$ for reaction between $Ce(CH_3COO)_3$ and $CeO_2{\cdot}2H_2O$
**3**	+1.089	+0.016	+0.204	–0.523
**4**	+0.825	+0.136	+0.357	–0.370
**5**	+0.455	+0.055	+0.276	–0.451
**6**	+0.210	–0.156	+0.064	–0.663
**7**	–0.019	–0.385	–0.164	–0.892

In summary, the cyclic voltammetry analyses show that, under the present experimental conditions, the formation of $Ce(OH)_{4(pt)}$ rather than $Ce(OH)_{4(aq)}$ occurs at pH ≥ 5, which allows the physical deposition of films. More importantly, although all of the ΔE_p are significantly >26 mV and so the the redox reactions are thermodynamically irreversible[9], the redox reactions can be made reversible through cathodic electrochemical reaction, resulting in reductive solubility of the as-deposited film to Ce^{3+} cations.

Supplementary Note 3. Characterisation of ultrathin CeO_{2-x} films

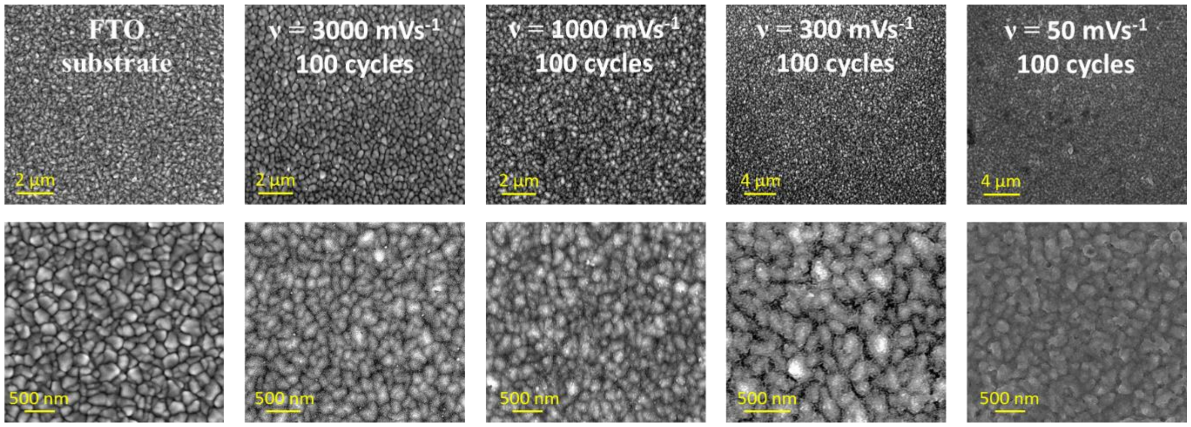

Supplementary Figure 4. SEM images of bare FTO substrate and CeO_{2-x} ultrathin films (100 cycles) deposited at scan rates of 3000, 1000, 300, and 50 mVs^{-1} (left-to-right sequence of figures is based on increasing thickness).

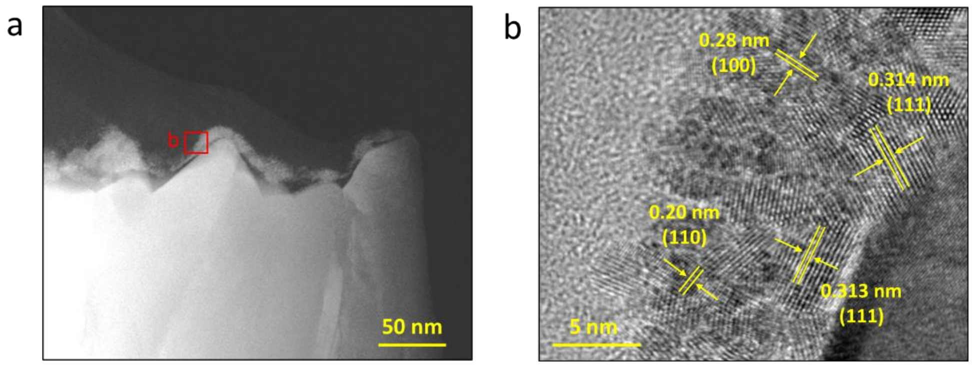

Supplementary Figure 5. (a) HAADF image, (b) HRTEM image of polycrystalline CeO_{2-x} film.

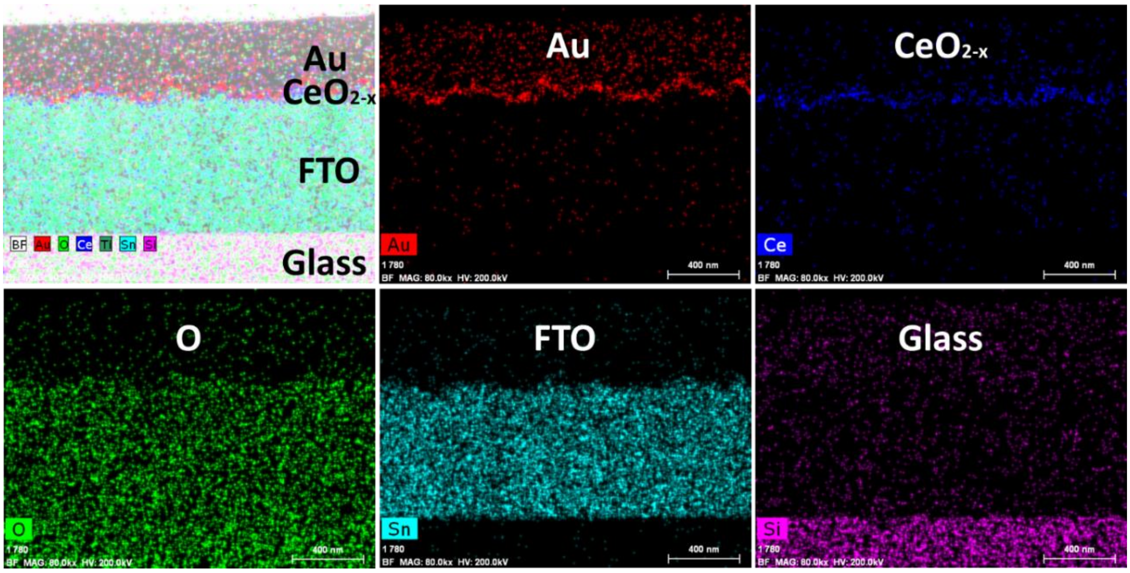

Supplementary Figure 6. EDS mapping of CeO_{2-x} ultrathin film deposited at υ = 3000 mVs^{-1}.

Supplementary Note 4. Quantitative XPS and EELS analyses

Quantitative XPS analysis: The Ce 3*d* spectra are composed of two multiplets corresponding the spin-orbit split for 3*d*5/2 (at lower binding energy) and 3*d*3/2 (at higher binding energy). The areas (A) under the two doublets peaks ~800 and 805 eV indicate the relative amounts of Ce^{3+} cations in CeO_{2-x}; the other three doublet peaks correspond to Ce^{4+}. Hence, the amount of Ce^{3+} is calculated by:

$$\frac{A[Ce^{3+}]}{A[Ce^{3+}] + A[Ce^{4+}]} \times 100 = Ce^{3+}\ at\% \qquad (1)$$

Similar calculations can be done using the O 1*s* spectra, although these must be deconvoluted to accommodate the contribution from physically adsorbed water.

EELS analysis:

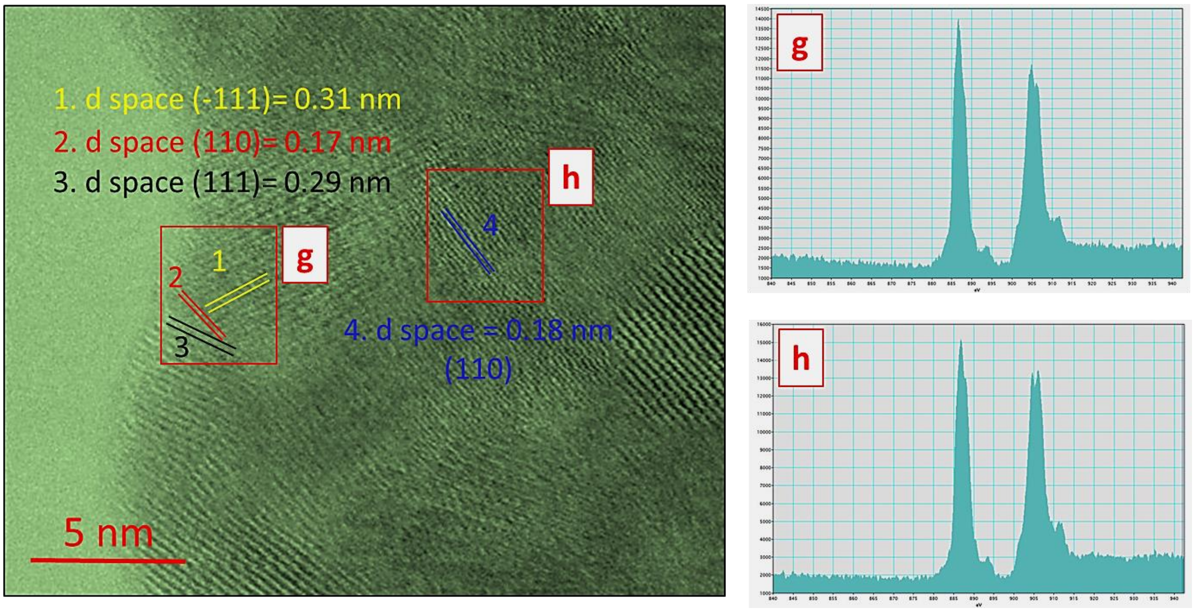

Supplementary Figure 7. HAADF images and corresponding EELS spectra form two representative areas of g (crystallite boundaries) and h (within crystallite).

Supplementary Note 5. Effect of pH on electrodeposition of CeO_{2-x}

The redox reactions were assessed further with cyclic voltammetry for 1-50 cycles at pH 4.0, 5.5, 6.0, as shown in Figure 8. At pH = 4.0 (Figure 8(a,b)), both the peak potentials and the voltammogram areas remained nearly constant during cycling; the area is indicative of the scale of the electrical charge associated with the reaction. This constancy indicates that no deposition occurred. The slight increase in the voltammogram areas (only observed for cathodic reduction), is derived from the electrochemical reduction of oxygen gas generated from unavoidable water oxidation. In contrast, at pH = 5.5 (Figure 8(c,d)), the voltammogram areas expanded asymmetrically up to cycle 30, where the area again reflects the charge storage associated with the volume of the deposition. Such expansion

also can be attributed to the deposition of the Ce(IV) species on the substrate. Further, the ΔE_p decreased from 530 mV for cycle 1 to 350 mV for cycle 30, which is attributed to increased electrical conductivity, after which ΔE_p becomes approximately constant. At pH = 6.0 (Figure 8(e,f)), the symmetry of the voltammogram areas remained unchanged up to cycle 10. Further cycling caused not only an expansion in the voltammogram areas but also the appearance of a pair of redox peaks at a lower $E_{1/2}$ of –0.02 V *vs.* Ag/AgCl.

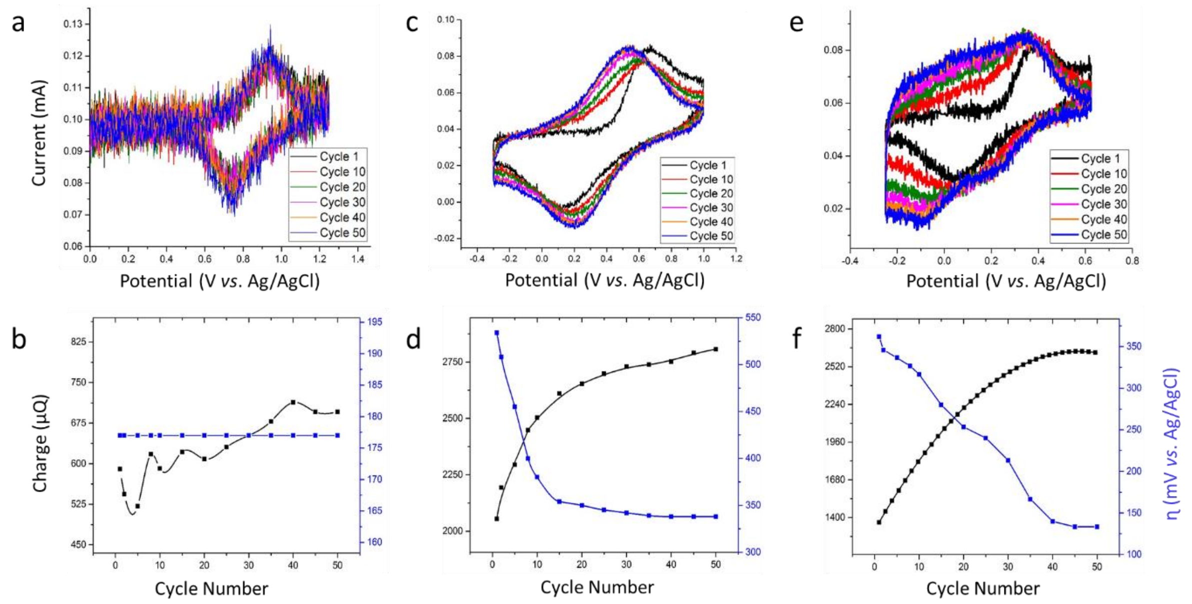

Supplementary Figure 8. Cyclic voltammetry analyses for Ce^{3+}-Ce^{4+}-CH_3COO-H_2O system for 1-50 cycles at υ = 100 mVs^{-1} at different pH. (a) 4.0, (c) 5.5, (e) 6.0; corresponding plots in (b), (d), and (f), respectively, of charge (Q) and overpotential (η) as a function of cycle number.

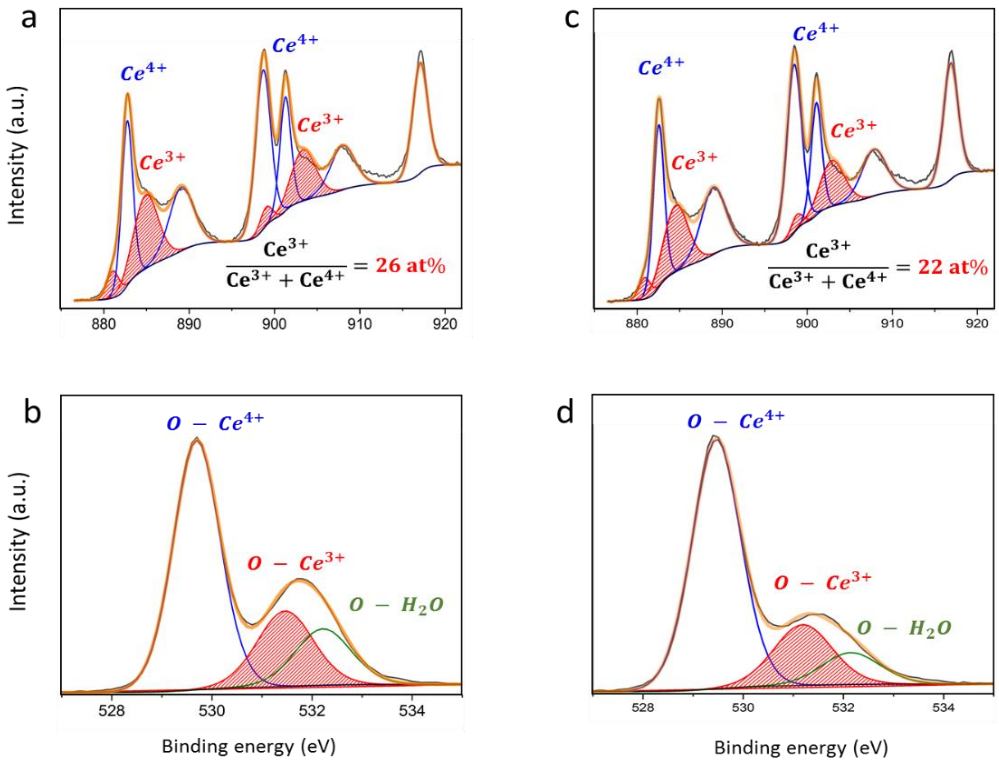

Supplementary Figure 9. Ce 3*d* XPS spectra for CeO_{2-x} ultrathin films electrodeposited at υ = 3000 mVs^{-1} with termination potentials: (a) –0.6 V, (c) +0.8 V; corresponding O 1*s* spectra in (b) and (d), respectively.

Supplementary Note 6. Chemical identification and calculation of peak areas observed in cyclic voltammetry deposition of CeO_{2-x} thin films

In this Supplementary Note, the chemical reactions associated with the redox peaks observed in cyclic voltammetry deposition of CeO_{2-x} thin films (Figure 2 in main text) are identified and discussed in details. The oxidation of Ce^{3+} (Ox_1) occurs at E = 0.47 V *vs* Ag/AgCl followed by reduction (Re_1) at E = –0.02 V *vs* Ag/AgCl. The accuracy of the identified peaks was confirmed over a wide range of pH values, the results of which are discussed comprehensively in Supplementary Note 1. Owing to the irreversibility of the redox reaction (Ox_1 and Re_1), as shown in Supplementary Note 1, some of the $Ce(OH)_4$ redissolves as soluble Ce^{3+} during reduction (Re_1), which is confirmed by the lower current density of Re_1 relative to that of Ox_1 ((anodic peak current)/(cathodic peak current) or I_{pa}/I_{pc} <1). These reactions and corresponding calculations are given in Table 5.

Supplementary Table 5. Chemical reactions attributed to $Ox_1 - Re_1$ peaks and their corresponding energy and potential calculations.

	$Ox_1 - Re_1$	Calculated ΔG^0(kJ/mol)	ΔG^0(kJ/mol)
**1**	$Ce^{4+} + e \rightarrow Ce^{3+}$	-168.20	$\Delta G^0_{Ce^{4+}} = -503.80$<br>$\Delta G^0_{Ce^{3+}} = -672.00$
**2**	$4H_2O \rightarrow 4H^+ + 4OH^-$	$+319.60$	$\text{Log } K_w = 14.00$
**3**	$Ce(OH)_{4(pt)} + 4H^+ \rightarrow Ce^{4+} + 4H_2O$	-0.02	$\Delta G^0_{Ce(OH)_4(pt)} = -1450.25$

**Total**	$Ce(OH)_{4(pt)} + e \Leftrightarrow Ce^{3+} + 4OH^-$	$E = -1.56 + 0.24\,p(OH) - 0.06 \log\frac{[Ce^{3+}]}{[Ce(OH)_{4(pt)}]}$<br>E (pH = 6) = +0.16 V *vs* Ag/AgCl<br>$E_{1/2}$ = +0.22 V *vs* Ag/AgCl

The remaining $Ce(OH)_4$ deposited on the substrate transforms rapidly to CeO_2 (as indicated in the Pourbaix diagram in Figure 3(d)). As given in Table 6, the reduction of as-prepared CeO_2 occurs at E = –0.13 V *vs* Ag/AgCl. This is followed by partial oxidation (annihilation of oxygen vacancies) by the following oxidation reaction (Ox_2) occurring at E = +0.07 V *vs* Ag/AgCl. This is further confirmed in Supplementary Note 7.

Supplementary Table 6. Chemical reactions attributed to $Ox_2 - Re_2$ peaks and their corresponding energy and potential calculations.

$Ox_2 - Re_2$		ΔG^0 (kJ/mol)
**1**	$CeO_2 + 2xe^- \rightarrow CeO_{2-x} + xO^{2-}$	$+ 165.00$
**2**	$O^{2-} + H^+ \rightarrow OH^-$	$- 157.20$
**3**	$CeO_2 + xH^+ + 2e^- \rightarrow CeO_{2-x} + xOH^-$	$E = +0.08 + 0.03\,p(OH) - 0.03\,pH - 0.03 \log\frac{[CeO_{2-x}]}{[CeO_2]}$<br>E (pH = 6) = – 0.06 V *vs* Ag/AgCl<br>$E_{1/2}$ = – 0.03 V *vs* Ag/AgCl

Owing to the formation of oxygen vacancies within the bulk of the deposited CeO_{2-x}, the insertion/disinsertion of protons commences during the reduction (Re_3) and oxidation (Ox_3) reactions, as given in Table 7. Although these phenomena have not been observed previously for CeO_2-based materials, they have been reported for a few metal oxides *(e.g.,* WO_3[11,12], RuO_2[13,14], and MnO_2[15]*)*.

Supplementary Table 7. Chemical reactions attributed to $Ox_3 - Re_3$ peaks and their corresponding potential calculations.

	$Ox_3 - Re_3$ (H^+ Intercalation/Disintercalation)
Re₃	$Ce^{4+}_{1-x}Ce^{3+}_{x}O_{2-y} + \alpha e^- + \beta H^+ \rightarrow Ce^{4+}_{1-(x+\alpha)}Ce^{3+}_{x+\alpha}O_{2-(y+\delta)}OH_{(\beta+y)}$
Ox₃	$Ce^{4+}_{1-(x+\alpha)}Ce^{3+}_{x+\alpha}O_{2-(y+\delta)}OH_{(\beta+y)} \rightarrow Ce^{4+}_{1-x}Ce^{3+}_{x}O_{2-y} + \alpha e^- + \beta H^+$

The charge contributions of each pair of reactions were calculated using Gaussian fitting. As can be seen from the corresponding values in Table 8, the contribution percentage of $\boldsymbol{Ox_1 - Re_1}$ decreases by increase the cycle number, while the actual values have insignificant decrease over cycling. Such phenomenon can be ascribed to continuous growth of the ultrathin film over cycling. Nonetheless, there are significant growth for both $\boldsymbol{Ox_2 - Re_2}$ and $\boldsymbol{Ox_3 - Re_3}$ by increase the cycle numbers. The growth in the peak area of $\boldsymbol{Ox_2 - Re_2}$ can be ascribed to the total increase in oxygen vacancy concentration formed within bulk of the ultrathin film. Consistent with thickening of the CeO_2 film

and increasing the amount of oxygen vacancies, the volume of hydrogen insertion/disinsertion reactions increase, resulting in having the highest areal contribution in the cyclic voltammogram.

Supplementary Table 8. Charge contribution and area calculation for identified peaks.

	Cycle 1	Cycle 25	Cycle 50
Redox reaction	$\boldsymbol{Ox_1 - Re_1}$ & $\boldsymbol{Re_2}$	$\boldsymbol{Ox_1 - Re_1, Ox_2 - Re_2, Ox_3 - Re_3}$	$\boldsymbol{Ox_1 - Re_1, Ox_2 - Re_2, Ox_3 - Re_3}$
Shared peak area for Ox_1/Re_1 (%)	29.4 x 10^{-5} C (77%)	26 x 10^{-5} C (25%)	20.9 x 10^{-5} C (16%)
Shared peak area for Ox_2/Re_2 (%)	8.6 x 10^{-5} C (23%) (only $\mathbf{Re_2}$)	38 x 10^{-5} C (37%)	44.9 x 10^{-5} C (36%)
Shared peak area for Ox_3/Re_3 (%)	0 x 10^{-5} C (0%)	37 x 10^{-5} C (38%)	55.8 x 10^{-5} C (48%)
Total peak area	38 x 10^{-5} C	101 x 10^{-5} C	122 x 10^{-5} C

Supplementary Note 7. Mechanism of electrodeposition of CeO_{2-x} (calculation of Gibbs free energy required for $V_O^{\bullet\bullet}$ formation (ΔG_{VO}))

The Gibbs free energy of oxygen vacancy formation (ΔG_{VO}) is calculated. The previously reported ΔG_{VO} is in the range +1.20-2.30 eV[10,16], depending on the grain size, exposed facets, and coordination number of the vacancy. The calculation in Table 9 shows that, despite the relatively high value, the ΔG_{VO} can be reduced significantly by delivering proton at the adjacent regions of as-deposited CeO_2 during cathodic reaction within aqueous environment. Thus the rapid interaction of protons and resultant O^{2-}, which is generated from the electroreduction of the CeO_2 film, facilitates the formation of $V_O^{\bullet\bullet}$ even within the bulk[17]. The required potential for ΔG_{VO} can be determined using the standard potential (E) given in Table 9. Considering the size of crystallites (3-8 nm), the best theoretical ΔG_{VO} was found to be 1.71 eV, which is the averaged ΔG_{VO} for surface, sub-surface, and bulk positions of $V_O^{\bullet\bullet}$[11,18]. For the $E_{1/2}$ in Figure 8(e) at pH = 6, ΔG_{VO} was measured to be –0.02 eV *vs.* Ag/AgCl, which is in good agreement with the calculated value of –0.06 eV *vs.* Ag/AgCl. According to the proposed mechanism, the formation of $V_O^{\bullet\bullet}$ is dependent on the pH of the aqueous solution, while increasing the pH reduce the required ΔG_{VO}.

Supplementary Table 9. Individual standard free energies of reaction (ΔG^0), overall standard potential equation (E), and Gibbs free energy of $V_O^{\bullet\bullet}$ formation (ΔG_{VO}).

No.	Reaction	ΔG^0 (eV)
**1**	$CeO_2 + 2xe^- \rightarrow CeO_{2-x} + xO^{2-}$	+1.71 (~1.20 to ~2.30)
**2**	$xO^{2-} + xH^+ \rightarrow xOH^-$	–1.63
**3**	$CeO_2 + xH^+ + 2e^- \rightarrow CeO_{2-x} + xOH^-$	$E = +0.08 + 0.03\,p(OH) - 0.03\,pH - 0.03\log\frac{[CeO_{2-x}]}{[CeO_2]}$
**Gibbs Free Energy of $V_O^{\bullet\bullet}$ Formation**		$\mathbf{\Delta G_{VO}\ (pH = 6) = -0.06\ eV}$ ***vs.*** **Ag/AgCl**

Since the formation of OH^- in an acidic environment might seem counter-intuitive, we have conducted further thermodynamic calculation. As shown in Table 10, oxygen vacancy formation in a relatively strong acidic environment (*e.g.*, pH ≤ 5.5) rich in hydrogen ions leads to the local formation of water[17]. In such a condition, the thermodynamic calculations (Table 10) show that the required Gibbs free energies (and corresponding $E_{1/2}$) for oxygen vacancy formation shift to values less than −1.26 eV *vs*. Ag/AgCl for pH ≤ 5.5. Such a high energy requirement for an aqueous system falls within the hydrogen-evolution region ($2H^+ + 2e \rightarrow H_2$), as confirmed by the corresponding Pourbaix diagram[1,3]. This is consistent with the cyclic voltammetry results (Figure 8c), where CeO_2 film deposition occurred at pH = 5.5 but there was no cyclic voltammetry peak from oxygen vacancy formation at values of approximately –0.02 eV *vs*. Ag/AgCl. Cycling at higher acidic pH values of 4.5 and 5.0 also showed the absence of an oxygen vacancy formation peak.

Supplementary Table 10. Thermodynamic calculation of oxygen vacancy formation reaction at different pH (5.0-5.8).

No.	Reaction	$\Delta G^0 (eV)$	
**1**	$CeO_2 + 2xe^- \rightarrow CeO_{2-x} + xO^{2-}$	+1.71 (~1.20 to ~2.30)	
**2**	$xO^{2-} + 2xH^+ \rightarrow xH_2O$	−2.45	
**3**	$CeO_2 + 2xH^+ + 2xe^- \rightarrow CeO_{2-x} + xH_2O$	$E = -0.74 - 0.06\ pH - 0.06 \log \frac{[CeO_{2-x}]}{[CeO_2]}$	
**Gibbs Free Energy of $V_O^{\bullet\bullet}$ Formation through H_2O Formation**		pH = 4.5	$\Delta G_{V_O} = -1.20$ eV
		pH = 5	$\Delta G_{V_O} = -1.23$ eV
		pH = 5.5	$\Delta G_{V_O} = -1.26$ eV

In contrast, as shown in Table 11, oxygen vacancy formation in a weakly acidic environment (*e.g.*, pH ≥ 6) less rich in hydrogen ions leads to the formation of OH^- ions, thus making the local environment essentially basic. In the presence of CeO_{2-x}, the amount of H^+ in basic solutions is much lower than acidic solutions, so the extent of reaction between as-produced O^{2-} with two H^+, leading to H_2O formation, is reduced significantly. Therefore, the formation of OH^- is favoured over that of H_2O[17], as confirmed by the calculations given in Table 11. This is consistent with the cyclic voltammetry results shown in Figure 8 (Supplementary) and Figure 2 (main text), where CeO_2 film deposition occurred at pH = 6.0 and was followed by the oxygen vacancy formation peak at $E_{1/2}$ = ~– 0.03 eV *vs*. Ag/AgCl. Cycling at less acidic (*i.e.*, more basic) pH values of 6.5 and 7.0 also showed the presence of an oxygen vacancy formation peak. In effect, a minimal pH of 6.0 is required in order to form an oxygen vacancy at accessible energies of approximately – 0.1 eV *vs*. Ag/AgCl, depending on pH of the environment.

Supplementary Table 11. Thermodynamic calculation of oxygen vacancy formation reaction at different pH (6.0-7.0).

No.	Reaction	$\Delta G^0 (eV)$

**4**	$CeO_2 + 2xe^- \rightarrow CeO_{2-x} + xO^{2-}$	+1.71 (~1.20 to ~2.30)
**5**	$xO^{2-} + xH^+ \rightarrow xOH^-$	−1.63
**6**	$CeO_2 + xH^+ + 2e^- \rightarrow CeO_{2-x} + xOH^-$	$E = +0.08 + 0.03\,p(OH) - 0.03\,pH - 0.03\log\frac{[CeO_{2-x}]}{[CeO_2]}$

**Gibbs Free Energy of $V_O^{\bullet\bullet}$ Formation through OH^- Formation**	pH = 6.0	$\Delta G_{V_O} = -0.06$ eV
	pH = 6.5	$\Delta G_{V_O} = -0.09$ eV
	pH = 7.0	$\Delta G_{V_O} = -0.12$ eV

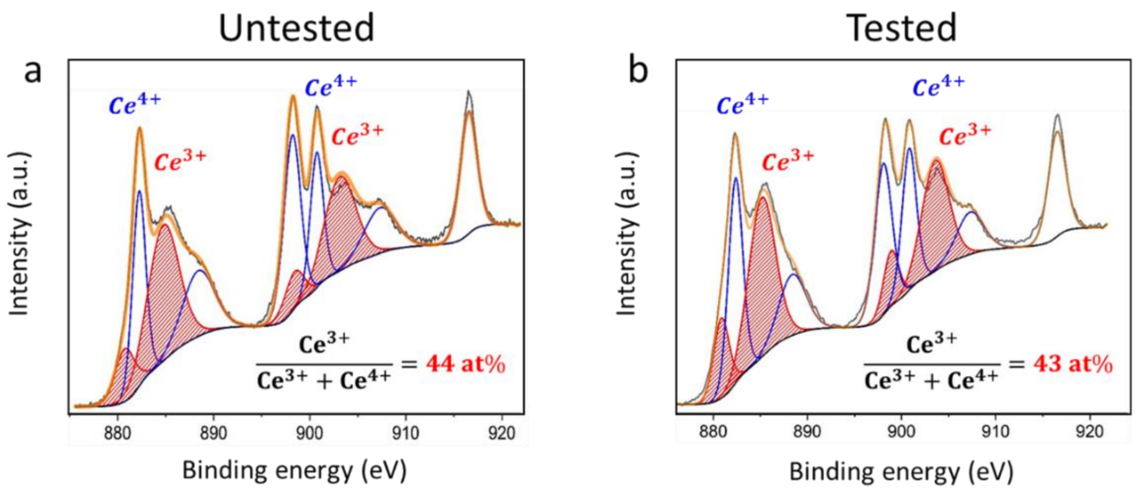

Supplementary Figure 10. Ce 3*d* XPS spectra for a single CeO_{2-x} ultrathin film electrodeposited at υ = 3000 mVs^{-1}. (a) untested, (b) after 1000 application cycles.

Supplementary Note 8. Energy storage applications

Charge Storage Analysis:

The volumetric and areal capacitances (C) are calculated using the following equation[4]:

$$C = \frac{\int_{E_1}^{E_2} i\,(E)\,dE}{2\,(E_2 - E_1)A\nu} \qquad (2)$$

E_1 and E_2 = Cut-off potentials (V)

$i(E)$ = Instantaneous current (A)

$\int_{E_1}^{E_2} i(E)\,dE$ = Total voltametric charge (integrated voltammagram area; C)

A = Electrode dimensions (length x width; cm^2)

V = Electrode dimensions (length x width x thickness, measured by TEM, AFM, TOFSIMS; cm^3)

N = Scan rate (VS^{-1})

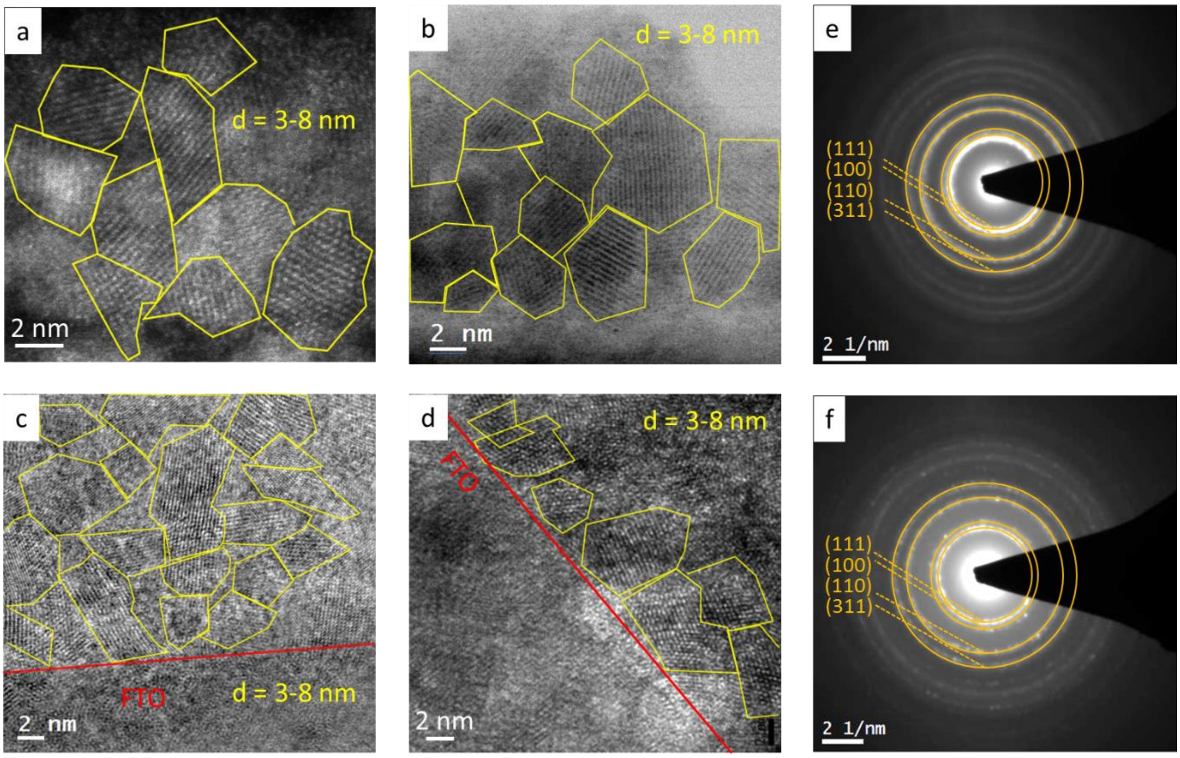

Supplementary Figure 11. Thin film deposited at different scan rates 50-3000 mVs^{-1}. (a,b) HAADF images of ultrathin films deposited at 3000 mVs^{-1} and 1000 mVs^{-1}, respectively. (c,d) HRTEM images, and (e,f) SAED patterns of ultrathin film deposited at 300 mVs^{-1} and 50 mVs^{-1}, respectively.

Supplementary Note 9. Effect of crystallite size on calculated band gap energies

It is possible that there may be nano-size effects on the estimation of the E_g in ceria, as suggested by atomic packing considerations of Ce-O bond lengths of nanoparticles[19]. However, such size effects cannot be simulated directly by DFT since the electronic band structure of a system requires periodicity in at least one dimension for the Bloch theorem to be valid and nanoparticles are zero-dimensional and hence lack periodicity. However, potential size effects on the E_g have been examined by the DFT calculations by considering 2% expansion or 2% contraction of the Ce-O bond lengths that correspond to analogous calculations based on the presence and absence of ligands in the growth environment[19]. The resultant DFT calculations for stoichiometric CeO_2 (Figure 12) reveal that 2% expansion or contraction in the Ce-O bond lengths leads to an almost negligible 2% reduction or 3% increase in the E_g, respectively. For non-stoichiometric CeO_{2-x}, 2% expansion or contraction results in only a 3% decrease or 2% increase in the E_g, respectively. In effect, if there is any size effect on the E_g corresponding to the preceding conditions[19], it is not significant.

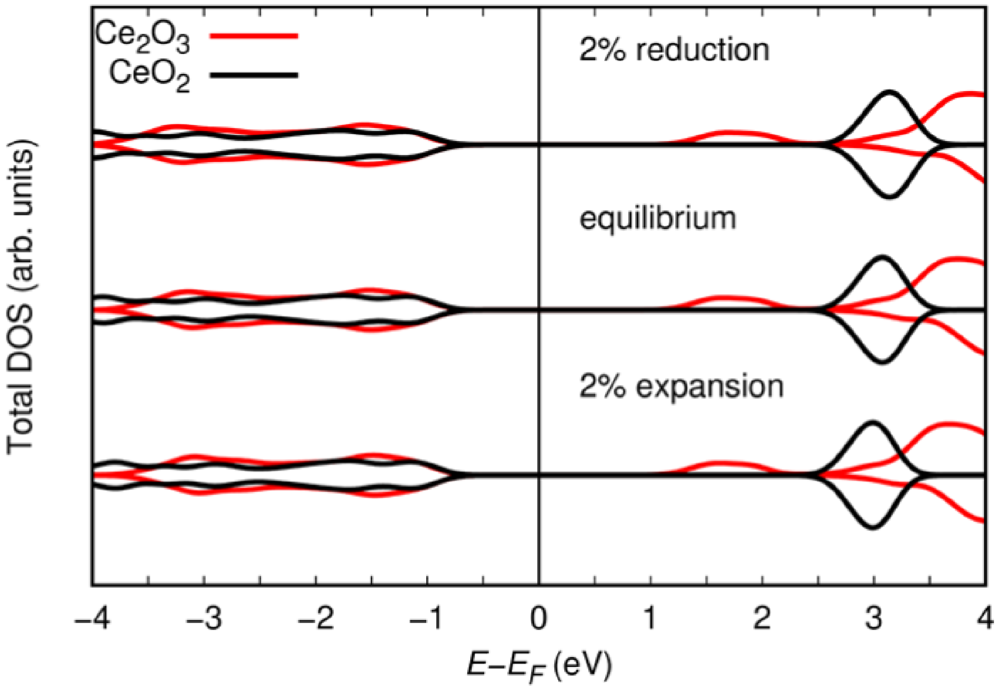

Supplementary Figure 12. Influence of potential nano-size effects on calculated energy band gap of stoichiometric and non-stoichiometric bulk ceria. Calculated spin-polarised total density of states showing spin-up (positive) and spin-down (negative) components; the Fermi energy level has been shifted to zero in all the cases.

Supplementary Note 10. Effect of scan rate on pseudocapacitive behaviour

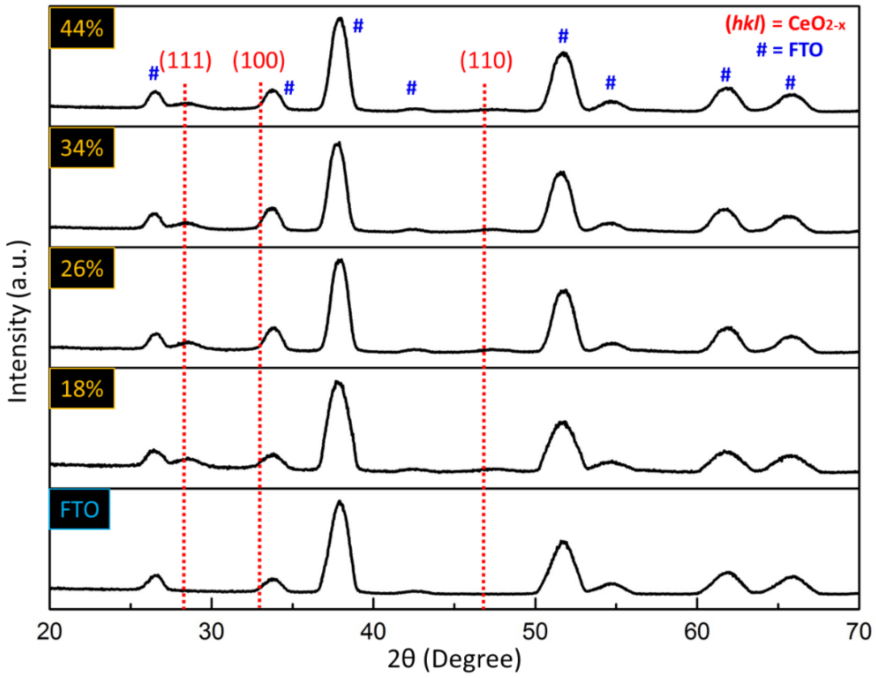

Supplementary Figure 13. XRD patterns for CeO_{2-x} ultrathin films and FTO substrate for [Ce^{3+}] = 18, 26, 34, 44 at% ((*hkl*) = CeO_{2-x}, **#** = FTO).

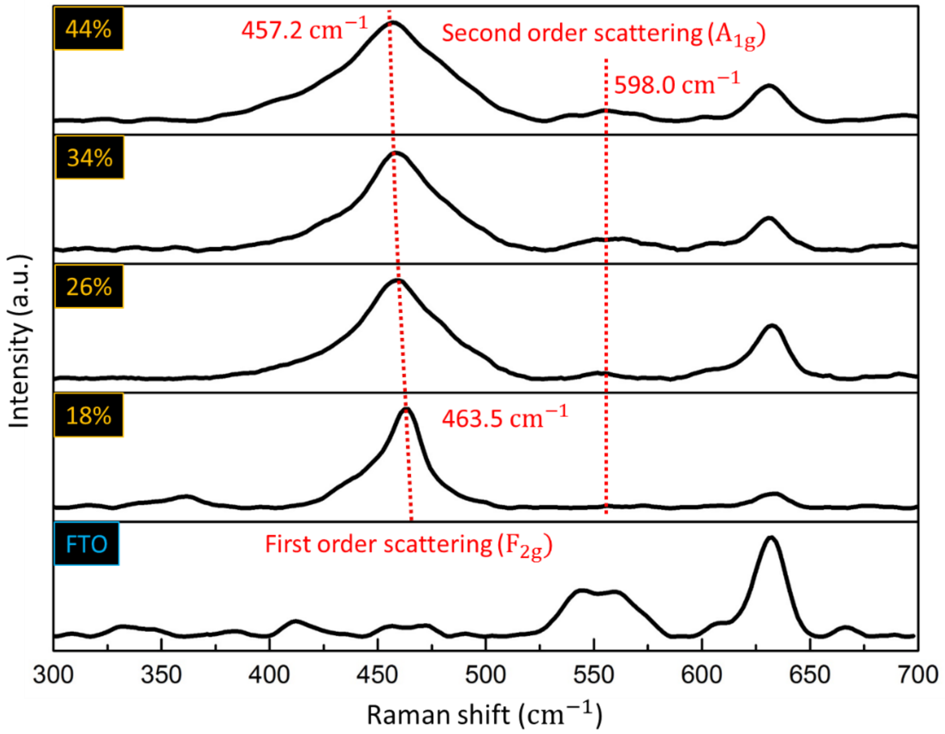

Supplementary Figure 14. Laser Raman microspectra for CeO_{2-x} ultrathin films and FTO substrate for [Ce^{3+}] = 18, 26, 34, 44 at% (F_{2g} = Stretching vibration of cerium coordinated with 8 oxygen atoms, A_{1g} = defect-induced vibrational mode (O^{2-})[20].

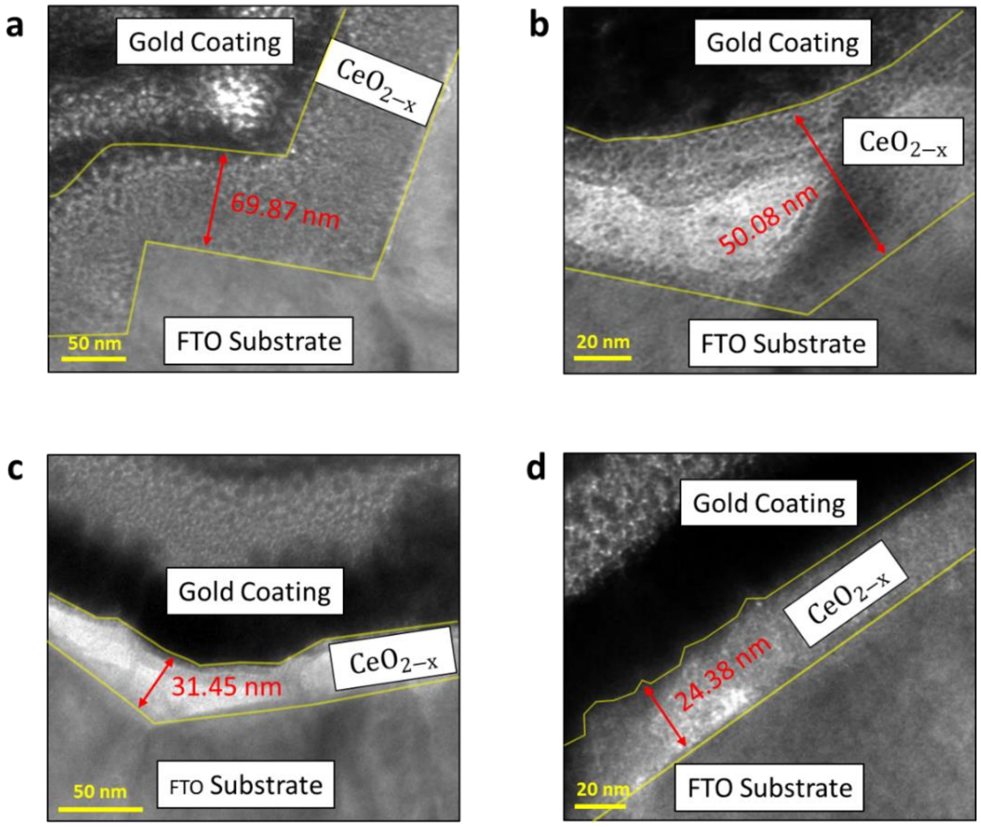

Supplementary Figure 15. Cross al TEM images of CeO_{2-x} ultrathin films electrodeposited for 100 cycles at different υ. (a) 50 mVs^{-1}, (b) 300 mVs^{-1}, (c) 1000 mVs^{-1}, (d) 3000 mVs^{-1}.

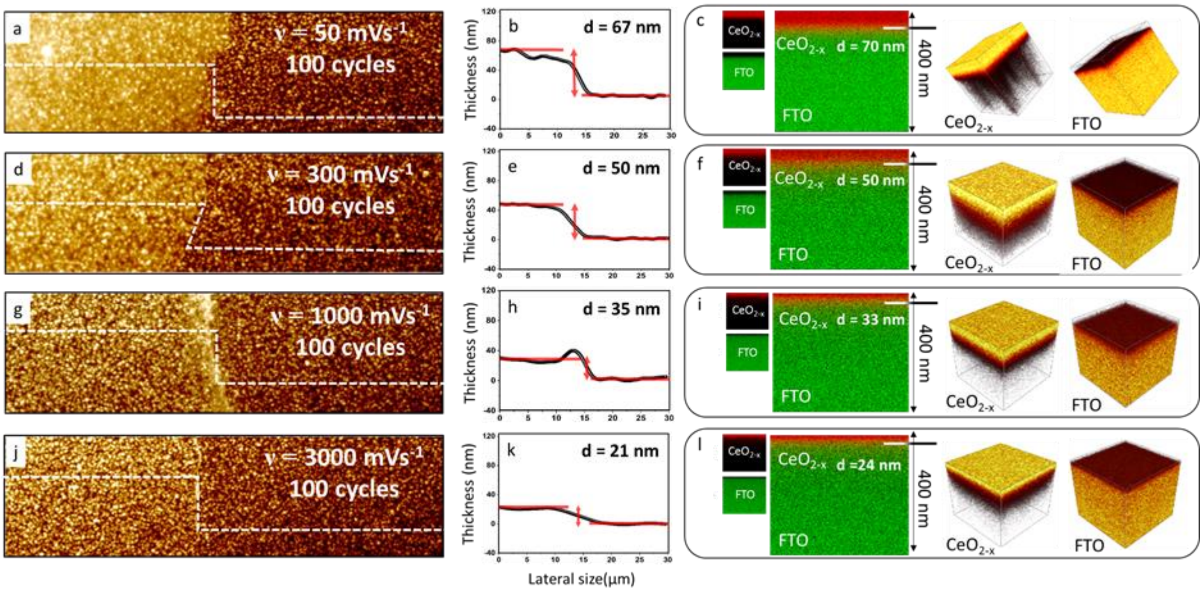

Supplementary Figure 16. Thicknesses of CeO_{2-x} ultrathin films synthesised at constant cycle number 100 and different scan rates: (a) 50, (d) 300 (g) 1000, (j) 3000 mVs^{-1}: (a, d, g, j) AFM images at cross sectional regions between ultrathin film and FTO substrate; (b,e,h,k) height profile plots of corresponding AFM images; (c,f,i,l) 2D and 3D SIMS elemental images indicating thicknesses of CeO_{2-x} ultrathin films.

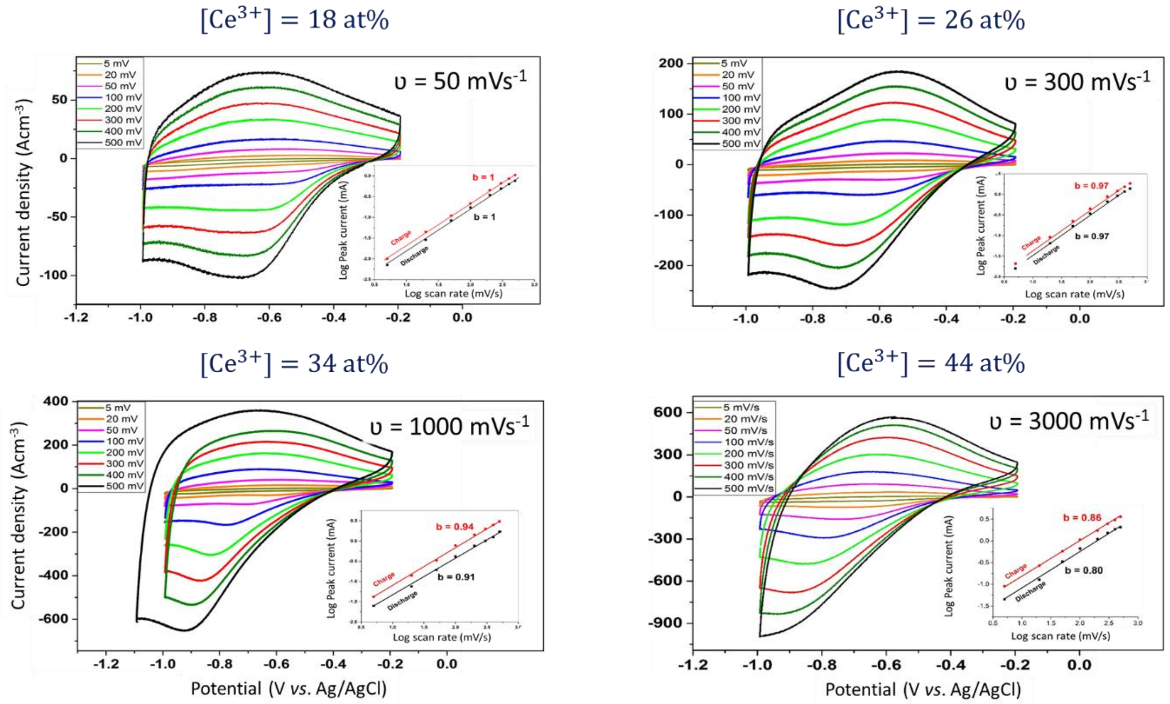

Supplementary Figure 17. Voltammograms and plots (insets) of log peak current (mA) *vs.* log υ (mVs^{-1}) for CeO_{2-x} ultrathin films for different [Ce^{3+}]. (a) 18, (b) 26, (c) 34, (d) 44 at%.

Photoluminescence spectroscopy (PL), which is used commonly to evaluate relative charge carrier concentrations ($Ce^{4+} \rightarrow Ce^{3+}$ redox, O vacancy formation, and electron/hole pair generation)[21,22], also was conducted in the present work in order to confirm that cyclic voltammetry deposition can be used to control defect concentrations in CeO_{2-x}. The emission intensity in the PL spectra derives from charge-carrier (electron/hole) recombination, thereby suggesting reduction in the concentrations of mobile defects[21,22] as the peak intensities increase.

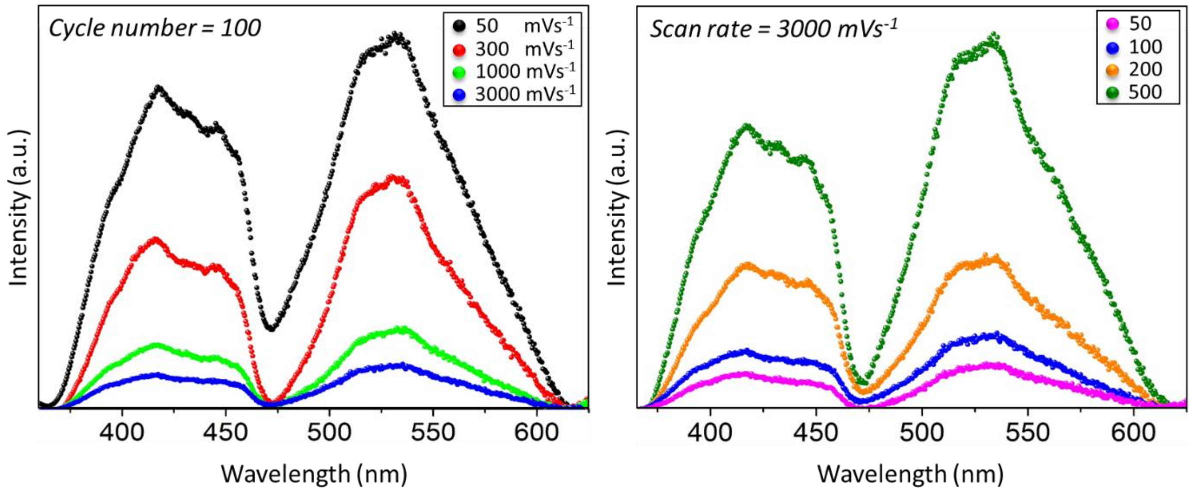

Supplementary Figure 18. PL spectra of ultrathin films prepared at different scan rates (100 cycles at 50-3000 mVs^{-1}) and cycle numbers (50-500 at 3000 mVs^{-1}).

Supplementary Note 11. Effect of cycle number on redox pseudocapacitive behaviour

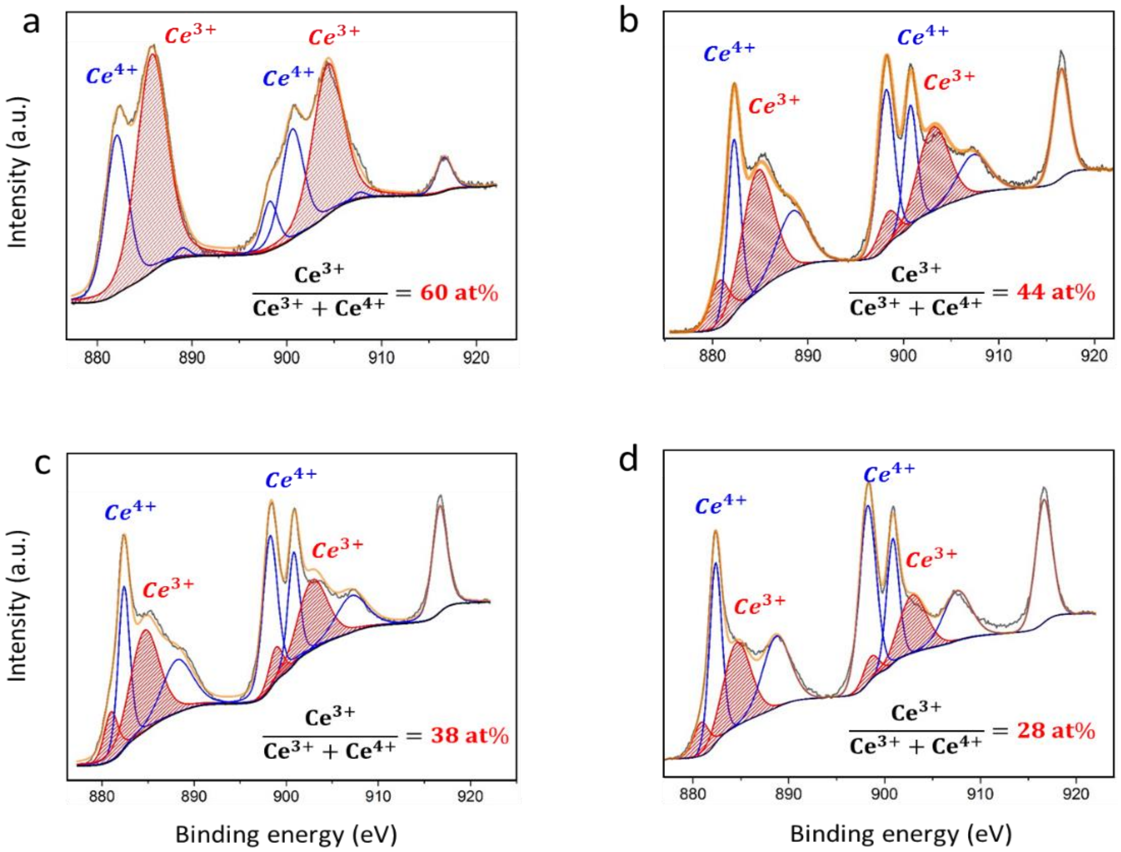

Supplementary Figure 19. Ce 3*d* XPS spectra for CeO_{2-x} ultrathin films electrodeposited at υ = 3000 mVs^{-1} for different cycle numbers. (a) 50, (b) 100, (c) 200, (d) 500.

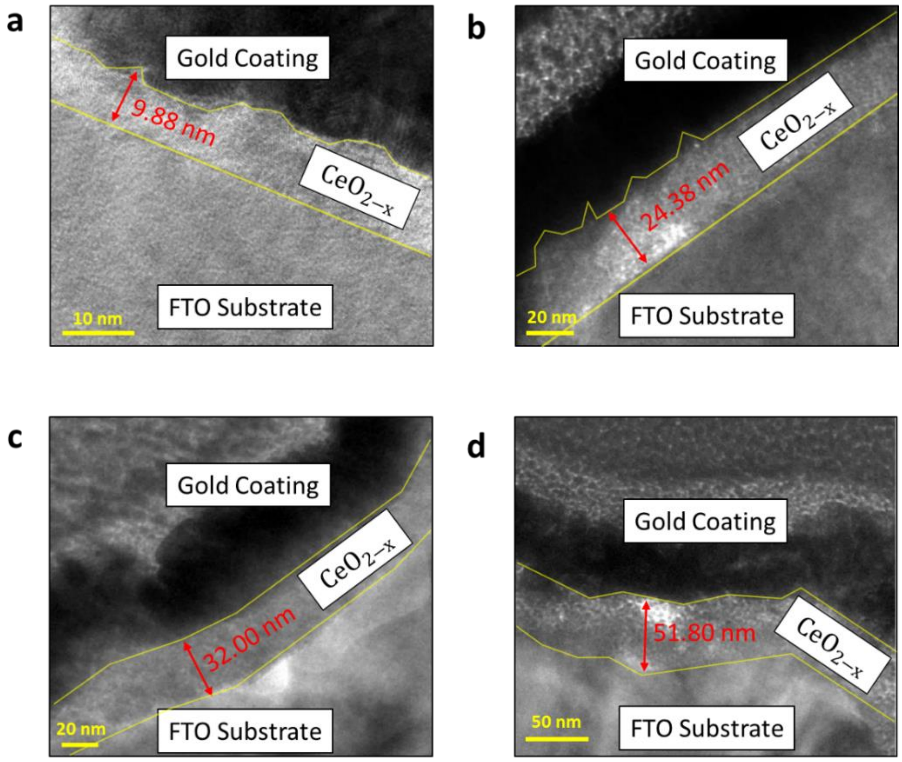

Supplementary Figure 20. Cross sectional TEM images for CeO_{2-x} ultrathin films electrodeposited at υ = 3000 mVs^{-1} for different cycle numbers. (a) 50, (b) 100 (c) 200, (d) 500.

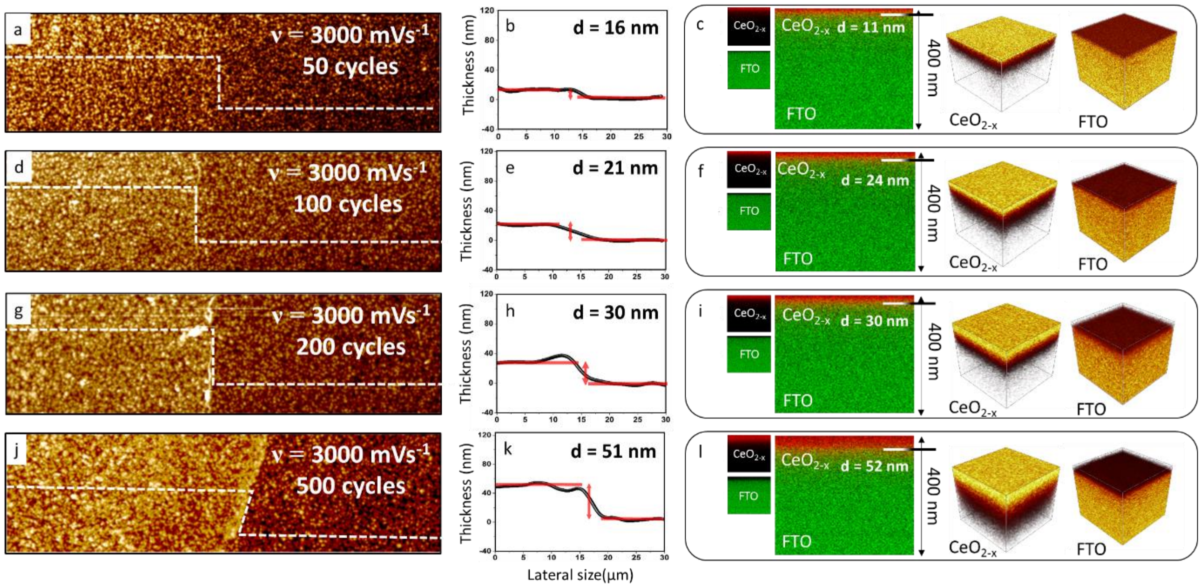

Supplementary Figure 21. Thicknesses of CeO_{2-x} ultrathin films synthesised at constant scan rate 3000 mVs^{-1} and different cycle numbers: (a) 50, (d) 100 (g) 200, (j) 500: (a, d, g, j) AFM images at cross sectional regions between ultrathin film and FTO substrate; (b,e,h,k) height profile plots of corresponding AFM images; (c,f,i,l) 2D and 3D SIMS elemental images indicating thicknesses of CeO_{2-x} ultrathin films.

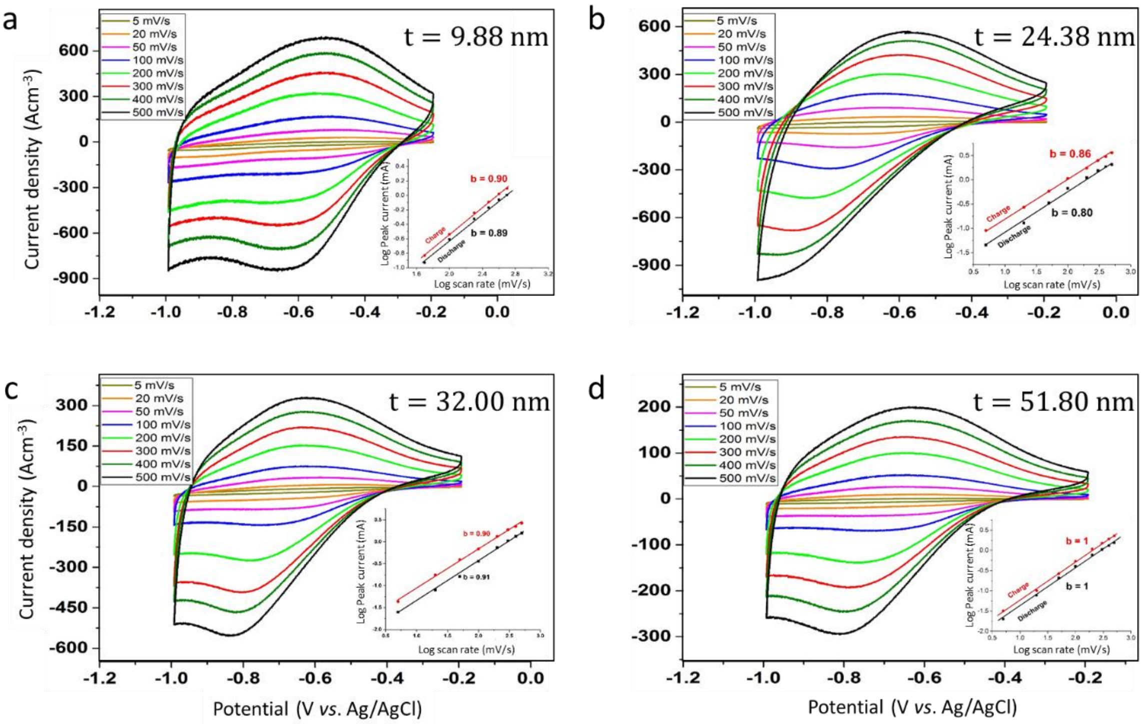

Supplementary Figure 22. Cyclic voltammograms and plots (insets) of log peak current (mA) *vs*. log υ (mVs^{-1}) for CeO_{2-x} ultrathin films electrodeposited at υ = 3000 mVs^{-1} for different cycle numbers. (a) 50, (b) 100, (c) 200, (d) 500. The corresponding Thicknesses (t) are shown in top right of the Figures.

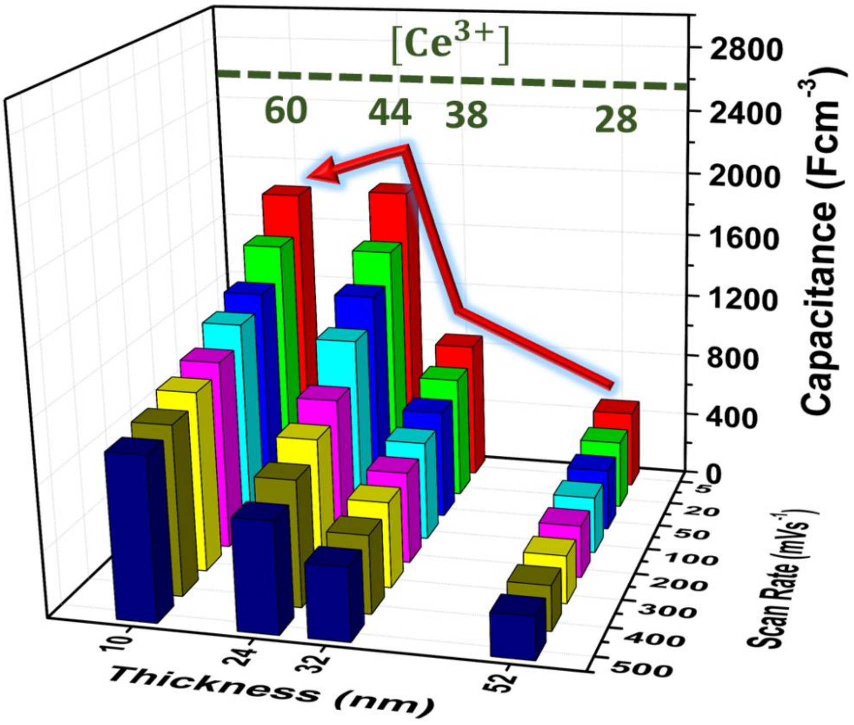

Supplementary Figure 23. Three-dimensional plot showing volumetric capacitances, scan rates, thicknesses, and [Ce^{3+}] for CeO_{2-x} ultrathin films.

Supplementary Note 12. Comparison with other work

Supplementary Table 12. Comparison of data for highly reported ultrathin-film pseudocapacitors.

Ultrathin Films	Volumetric Capacitance (Fcm^{-3})	Areal Capacitance ($mFcm^{-2}$)	Thickness (nm)	Ref
**CeO_{2-x}**	**1873**	**4.56**	**24**	**This work**
MnO_2/Au	1160	-	50	[23]
$VOPO_4$	929	8.36	90	[24]
$Ni(OH)_2$	661	3.30	50	[25]
VS_2	317	4.70	150	[26]
MnO_2/Graphene	250	400	?	[27]
MnO_2/MWNT	246	-	240	[28]
MoO_3		0.06	500	[29]
RuO_2/PEDOT	190	1.2	35-100	[30]

Supplementary Note 13. Computational methodology for calculation of $Ce(OH)_4$ Gibbs free energy

All geometry optimizations and vibrational analyses were performed with the Gaussian03 code[31] for a $Ce(OH)_4$ cluster to determine proximity of energies compared to the linear free energy relationship approach. For consistency, optimised structures and force fields were calculated at the CAM-B3LYP level of theory[32] with a Pople-style 6-31+g* basis set[33-35] for O-H species whilst for Ce, relativistic MWB ECPs (MWB48) were employed[36]. To overcome challenge of convergence, the extremely efficient quadratically convergent (QC) SCF procedure[37] was applied. All solution phase vibrational data were calculated from solution phase optimized structures using gas-phase geometries as a starting point incorporating the SCRF continuum solvation method[38]. The default dielectric constant for water, ε = 78.39, was used for all calculations. All energies include zero-point energy and an enthalpy correction (from 0 to 298.15 K). Calculated and experimental thermal energies were obtained from the work of Pourbaix[39] whereas free energies for O-H species and Ce species at 298 K were extracted from JANEF tables[40] and that of the reference of Lange's Handbook of Chemistry[41].

Supplementary References

1 Hayes, S. A. Yu, P. O'Keefe, T.J. O'Keefe, M.J. & Stoffer, J.O. The phase stability of cerium species in aqueous systems. I. E-pH diagram for the Ce-$HClO_4$-H_2O system. *J. Electrochem. Soc.* **149**, C623-C630, doi:10.1149/1.1516775 (2002).

2 Chirkst, D.E., Lobacheva, O.L. & Berlinskii, I.V. Gibbs energies of formation of hydroxides of lanthanides and yttrium. *Russ. J. Phys. Chem. A.* **84**, 2047-2050, doi:10.1134/S003602441012006x (2010).

3 Channei, D. *et al.* Aqueous and surface chemistries of photocatalytic Fe-doped CeO_2 nanoparticles. *Catalysts.* **7**, doi:ARTN 45, 10.3390/catal7020045 (2017).

4 Chen, W. Fan, Z. L. Gu, L. Bao, X. H. & Wang, C. L. Enhanced capacitance of manganese oxide via confinement inside carbon nanotubes. *Chem. Comm.* **46**, 3905-3907, doi:10.1039/c000517g (2010).

5 Kulp, E. A. Limmer, S. J. Bohannan, E. W. & Switzer, J. A. Electrodeposition of nanometer-thick ceria films by oxidation of cerium (III)-acetate. *Solid. State. Ionics.* **178**, 749-757, doi:10.1016/j.ssi.2007.03.012 (2007).

6 Bouchaud, B. Balmain, J. Bonnet, G. & Pedraza, F. pH-distribution of cerium species in aqueous systems. *J. Rare. Earth.* **30**, 559-562, doi:10.1016/S1002-0721(12)60091-X (2012).

7 Xu, H. Wang, Y. & L. Barton, L. Application of a linear free energy relationship to crystalline solids of MO_2 and $M(OH)_4$. *J. Nucl. Mater.* **273**, 343-346, doi:https://doi.org/10.1016/S0022-3115(99)00092-6 (1999).

8 Zhu, N. Han, S. Gan, S. Ulstrup, J. & Chi, Q. Graphene paper doped with chemically compatible prussian blue nanoparticles as nanohybrid electrocatalyst. *Adv. Funct. Mater.* **23**, 5297-5306, doi:10.1002/adfm.201300605 (2013).

9 Bockris, J. O. M. Reddy, A. K. N. & Gamboa-Aldeco, M. E. *Modern Electrochemistry 2A: Fundamentals of Electrodics*. (Springer US, 2007).

10 Pesterfield, L. L. Maddox, J. B. Crocker, M. S. & Schweitzer, G. K. Pourbaix (E-pH-M) diagrams in three dimensions. *J. Chem. Educ.* **89**, 891-899, doi:10.1021/ed200423n (2012).

11 Zhu, M. Meng, W. Huang, Y. Huang, Y. & Zhi, C. Proton-insertion-enhanced pseudocapacitance based on the assembly structure of tungsten oxide. *ACS Appl. Mater. Interfaces.* **6**, 18901-18910, doi:10.1021/am504756u (2014).

12 Kim, D.-J. Pyun, S.-I. & Choi, Y.-M. A study on the hydrogen intercalation into rf-magnetron sputtered amorphous WO_3 film using cyclic voltammetry combined with electrochemical quartz crystal microbalance technique. *Solid State Ionics.* **109**, 81-87, doi:https://doi.org/10.1016/S0167-2738(98)00099-X (1998).

13 Chen, L. Y. *et al.* Toward the theoretical capacitance of RuO_2 reinforced by highly conductive nanoporous gold. *Adv. Energy Mater.* **3**, 851-856, doi:10.1002/aenm.201300024 (2013).

14 Kim, I.-H. & Kim, K.-B. Electrochemical characterization of hydrous ruthenium oxide thin-film electrodes for electrochemical capacitor applications. *J. Electrochem. Soc.* **153**, A383-A389, doi:10.1149/1.2147406 (2006).

15 Long, J. W. *et al.* Asymmetric electrochemical capacitors—Stretching the limits of aqueous electrolytes. *MRS Bulletin* **36**, 513-522, doi:10.1557/mrs.2011.137 (2011).

16 Migani, A., Vayssilov, G. N., Bromley, S. T., Illas, F. & Neyman, K. M. Greatly facilitated oxygen vacancy formation in ceria nanocrystallites. *Chem. Comm.* **46**, 5936-5938, doi:10.1039/c0cc01091j (2010).

17 Close, T. Tulsyan, G. Diaz, C. A. Weinstein, S. J. & Richter, C. Reversible oxygen scavenging at room temperature using electrochemically reduced titanium oxide nanotubes. *Nat. NaNotechnol.* **10**, 418-422, doi:10.1038/Nnano.2015.51 (2015).

18 Migani, A. Vayssilov, G. N. Bromley, S. T. Illas, F. & Neyman, K. M. Dramatic reduction of the oxygen vacancy formation energy in ceria particles: a possible key to their remarkable reactivity at the nanoscale. *J. Mater. Chem..* **20**, 10535-10546, doi:10.1039/C0JM01908A (2010).

19 Sun, C. & Xue, D. Size-dependent oxygen storage ability of nano-sized ceria. *Phys. Chem. Chem. Phys.* **15**, 14414-14419, doi:10.1039/C3CP51959G (2013).

20 Wu, Z. L. Li, M. J. Howe, J. Meyer, H. M. & Overbury, S. H. Probing defect sites on CeO_2 nanocrystals with well-defined surface planes by raman spectroscopy and O_2 adsorption. *Langmuir.* **26**, 16595-16606, doi:10.1021/la101723w (2010).

21 Channei, D. *et al.* Photocatalytic degradation of methyl orange by CeO_2 and Fe–doped CeO_2 films under visible light irradiation. *Sci. Rep.* **4**, 5757, doi:10.1038/srep05757 (2014).

22 Tamizhdurai, P. *et al.* Environmentally friendly synthesis of CeO_2 nanoparticles for the catalytic oxidation of benzyl alcohol to benzaldehyde and selective detection of nitrite. *Sci. Rep.* **7**, 46372, doi:10.1038/srep46372 (2017).

23 Lang, X. Y. Hirata, A. Fujita, T. & Chen, M. W. Nanoporous metal/oxide hybrid electrodes for electrochemical supercapacitors. *Nat. NaNotechnol.* **6**, 232-236, doi:10.1038/nnano.2011.13 (2011).

24 Wu, C. Z. *et al.* Two-dimensional vanadyl phosphate ultrathin nanosheets for high energy density and flexible pseudocapacitors. *Nat. Commun.* **4**, doi:ARTN 243110.1038/ncomms3431 (2013).

25 Xie, J. F. *et al.* Layer-by-layer beta-Ni$(OH)_2$/graphene nanohybrids for ultraflexible all-solid-state thin-film supercapacitors with high electrochemical performance. *Nano. Energy.* **2**, 65-74, doi:10.1016/j.nanoen.2012.07.016 (2013).

26 Feng, J. *et al.* Metallic Few-Layered VS_2 Ultrathin nanosheets: high two-dimensional conductivity for in-plane supercapacitors. *J. Am. Chem. Soc.* **133**, 17832-17838, doi:10.1021/ja207176c (2011).

27 Peng, L. L. *et al.* Ultrathin two-dimensional MnO_2/graphene hybrid nanostructures for high-performance, flexible planar supercapacitors. *Nano. Lett.* **13**, 2151-2157, doi:10.1021/nl400600x (2013).

28 Lee, S. W. Kim, J. Chen, S. Hammond, P. T. & Shao-Horn, Y. Carbon nanotube/manganese oxide ultrathin film electrodes for electrochemical capacitors. *Acs. Nano.* **4**, 3889-3896, doi:10.1021/nn100681d (2010).

29 Mendoza-Sanchez, B. Brousse, T. Ramirez-Castro, C. Nicolosi, V. & Grant, P. S. An investigation of nanostructured thin film alpha-MoO_3 based supercapacitor electrodes in an aqueous electrolyte. *Electrochim. Acta.* **91**, 253-260, doi:10.1016/j.electacta.2012.11.127 (2013).

30 Lu, K. *et al.* Cation intercalation in manganese oxide nanosheets: effects on lithium and sodium storage. *Angew. Chem. Int. Edit.* **55**, 10448-10452, doi:10.1002/anie.201605102 (2016).

31 Gaussian v. G03 (Gaussian, Inc., Wallingford, CT, 2003).

32 Yanai, T. Tew, D. P. & Handy, N. C. A new hybrid exchange–correlation functional using the Coulomb-attenuating method (CAM-B3LYP). *Chemical. Physics. Letters.* **393**, 51-57, doi:10.1016/j.cplett.2004.06.011 (2004).

33 Pople, J. A. Nobel lecture: quantum chemical models. *Reviews of Modern Physics.* **71**, 1267-1274, doi:10.1103/RevModPhys.71.1267 (1999).

34 Petersson, G. A. *et al.* A complete basis set model chemistry. I. The total energies of closed-shell atoms and hydrides of the first-row elements. *The Journal of Chemical Physics.* **89**, 2193-2218, doi:10.1063/1.455064 (1988).

35 Petersson, G. A. & Al-Laham, M. A. A complete basis set model chemistry. II. Open-shell systems and the total energies of the first-row atoms. *The Journal of Chemical Physics.* **94**, 6081-6090, doi:10.1063/1.460447 (1991).

36 Andrae, D. Häußermann, U. Dolg, M. Stoll, H. & Preuß, H. Energy-adjusted ab initio pseudopotentials for the second and third row transition elements. *Theoretica. chimica. acta.* **77**, 123-141, doi:10.1007/BF01114537 (1990).

37 Bacskay, G. B. A quadratically convergent hartree-fock (QC-SCF) method. application to closed shell systems. *Chemical. Physics.* **61**, 385-404, doi:https://doi.org/10.1016/0301-0104(81)85156-7 (1981).

38 Scalmani, G. & Frisch, M. J. Continuous surface charge polarizable continuum models of solvation. I. General formalism. *J. Chem. Phys.* **132**, 114110, doi:10.1063/1.3359469 (2010).
39 Pourbaix, M. *Atlas of electrochemical equilibria in aqueous solutions*. p551 (National Association of Corrosion Engineers, 1974).
40 NIST. *NIST-JANAF Thermochemical Tables* <https://janaf.nist.gov/> (1998).
41 Dean, J. A. *Lange's Handbook of Chemistry*. Vol. 12th Edition (1979).
